# Short term cardiovascular symptoms improvement after deep brain stimulation in patients with Parkinson's disease: a systematic review

**DOI:** 10.1007/s00415-024-12459-1

**Published:** 2024-05-29

**Authors:** Francescopaolo Cucinotta, Bart Swinnen, Elena Makovac, Stephanie Hirschbichler, Erlick Pereira, Simon Little, Francesca Morgante, Lucia Ricciardi

**Affiliations:** 1 Department of Clinical and Experimental Medicine, University of Messina, Messina, Italy; 2 Neurosciences and Cell Biology Institute, Neuromodulation and Motor Control Section, St George’s University of London, London, UK; 3 UCSF, Department of Neurology, University of California San Francisco, San Francisco, CA USA; 4 UCSF, Weill Institute for Neurosciences, Movement Disorders and Neuromodulation Centre, University of California San Francisco, San Francisco, CA USA; 5 Department of Neurology and Clinical Neurophysiology, Amsterdam Neuroscience, Amsterdam University Medical Centers, University of Amsterdam, Amsterdam, The Netherlands; 6 Centre for Neuroimaging Science, King’s College, London, UK; 7 Brunel University London, Uxbridge, UK; 8 Karl Landsteiner University of Health Sciences, Dr. Karl-Dorrek-Straße 30, 3500 Krems, Austria; 9 Department of Neurology, University Hospital St. Pölten, Dunant-Platz 1, 3100 St. Pölten, Austria

**Keywords:** Deep brain stimulation, Parkinson’s disease, Cardiovascular functions, Heart rate variability

## Abstract

**Background:**

Autonomic dysfunction is common and disabling in Parkinson's disease (PD). The effects of deep brain stimulation (DBS) on the cardiovascular system in PD remain poorly understood. We aimed to assess the effect of DBS on cardiovascular symptoms and objective measures in PD patients.

**Methods:**

We conducted a systematic literature search in PubMed/MEDLINE.

**Results:**

36 out of 472 studies were included, mostly involving DBS of the subthalamic nucleus, and to a lesser extent the globus pallidus pars interna and pedunculopontine nucleus. Seventeen studies evaluated the effect of DBS on patient-reported or clinician-rated cardiovascular symptoms, showing an improvement in the first year after surgery but not with longer-term follow-up. DBS has no clear direct effects on blood pressure during an orthostatic challenge (n = 10 studies). DBS has inconsistent effects on heart rate variability (n = 10 studies).

**Conclusion:**

Current evidence on the impact of DBS on cardiovascular functions in PD is inconclusive. DBS may offer short-term improvement of cardiovascular symptoms in PD, particularly orthostatic hypotension, which may be attributed to dopaminergic medication reduction after surgery. There is insufficient evidence to draw conclusions on the direct effect of DBS on blood pressure and heart rate variability.

## Introduction

Dysfunction of the autonomic nervous system is common in people with Parkinson's disease (PD) and includes symptoms of altered cardiovascular, gastrointestinal, urinary, thermoregulatory, and sexual functions. Autonomic dysfunction in PD is an important determinant of disability, disease-related quality of life, and mortality [1]. The exact mechanisms underlying autonomic dysfunction in PD remain unclear, with varying roles attributed to degeneration of the central (e.g. preganglionic neurons within brainstem nuclei like the dorsal motor nucleus of the vagus nerve) and peripheral (e.g. postganglionic neurons and the enteric nervous system) autonomic nervous system [2], and more recently with changes in the brain regions regulating cardiovagal outflow (known as central autonomic network) [3, 4].

**Fig. 1:**
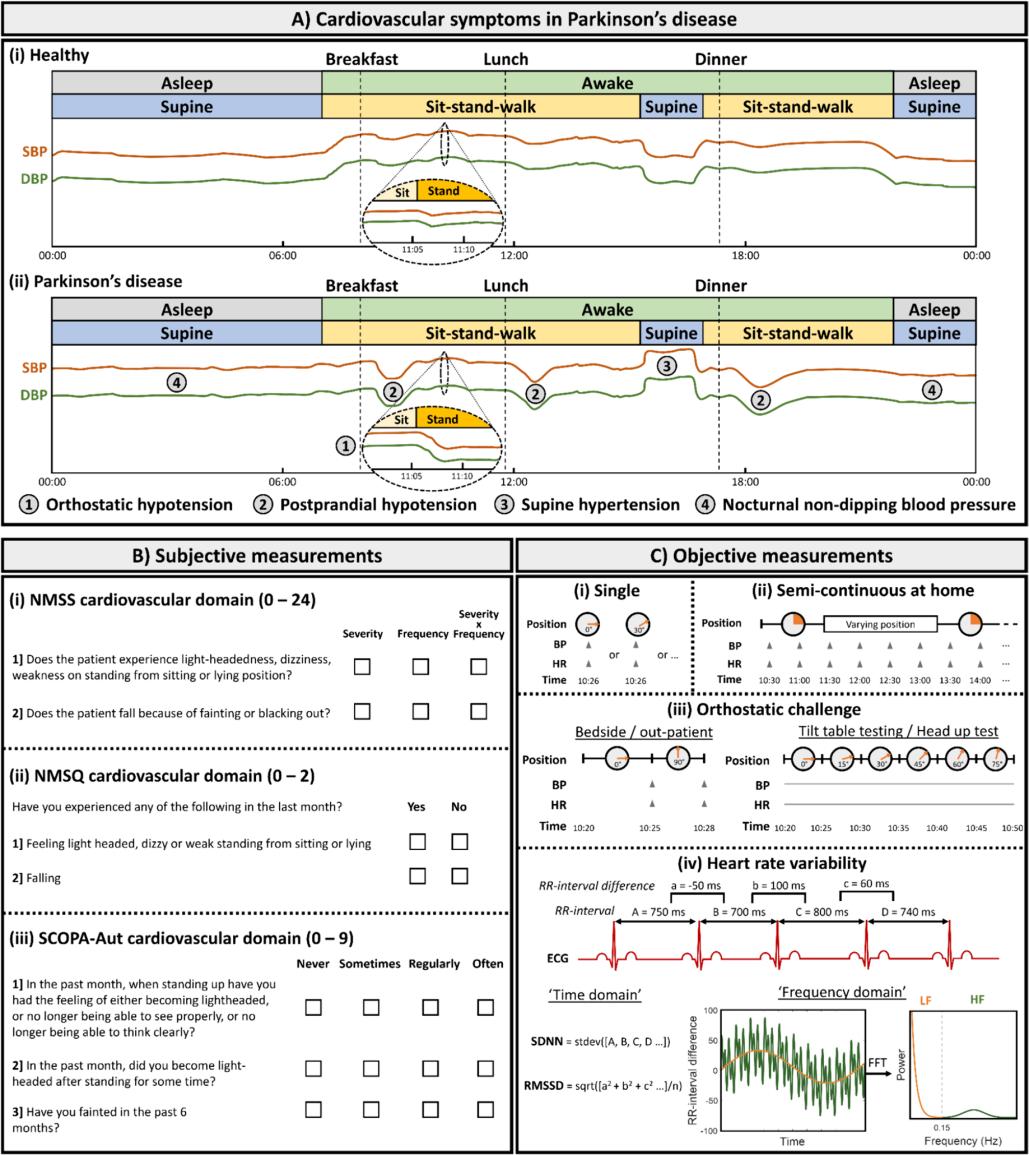
Phenomenology and assessment of cardiovascular symptoms in Parkinson’s disease. Panel (**A**) depicts cardiovascular symptoms in people with Parkinson’s disease (ii) compared to healthy individuals (i) as illustrated by a fictional 24-h blood pressure measurement. Systolic blood pressure (SBP) is indicated by the orange line, and diastolic blood pressure (DBP) by the green line. Wakefulness (i.e. awake versus asleep) and bodily positions/activities (i.e. supine versus sitting/standing/walking) are indicated by color-coded bars above each grach. Meal timepoints (i.e. breakfast, lunch, and dinner) are indicated by vertical dashed lines. The oval inserts depict a magnified curve of the blood pressure at the moment of transition from sitting to standing (i.e. an orthostatic challenge). The occurrence of cardiovascular symptoms in Parkinson’s disease are indicated by numbers within a circle. These are (1) orthostatic hypotension, (2) postprandial hypotension, (3) supine hypertension, and (4) nocturnal non-dipping blood pressure. Panel (**B**) depicts the three most frequently used questionnaires/scales to subjectively measure cardiovascular symptoms in people with Parkinson’s disease. These are (i) Non-Motor Symptoms Scale (NMSS), (ii) Non-Motor Symptoms Questionnaire (NMSQ), and (iii) Scales for Outcomes in Parkinson’s Disease—Autonomic (SCOPA-Aut). Panel (**C**) depicts objective measurements used to assess cardiovascular function in Parkinsons’s disease. These include: (i) single measurements of blood pressure (BP) and heart rate (HR) in the lying or sitting position, (ii) semi-continuous measurements of BP and HR at home, (iii) BP and HR measurements during an orthostatic challenge—either out-patient/bedside or with formal tilt table testing, and (iv) measures of heart rate variability (HRV) based on electrocardiography (ECG). HRV measures are derivations from the interbeat intervals (RR-interval), both in the time domain (e.g. Standard Deviation of the N–N intervals (SDNN) and Root Mean Square of Successive Differences (RMSSD)) and frequency domain (e.g. low frequency (LF) and high frequency (HF) resulting from a fast Fourier transformation (FFT))

Autonomic disturbances in PD can occur at any disease stage, even preceding the onset of motor symptoms—coined ‘prodromal’ symptoms [5]. Overall, cardiovascular symptoms occur in approximately 70% of PD patients [6]. They can be present at any stage of the disease—including early in the disease course [7], and symptoms include orthostatic hypotension, postprandial hypotension, supine hypertension, and nocturnal non-dipping blood pressure (Fig. 1A) [2]. Although these symptoms may often go unnoticed by the patient, they are associated with important and considerable risks. Orthostatic hypotension can be the cause of unexplained falls, and supine or nocturnal hypertension can lead to potentially fatal renal and cerebral vasculopathies [8, 9]. Both parasympathetic [10] and sympathetic [11] nervous system dysfunction have been suggested to contribute to these cardiovascular symptoms. Since sympathetic denervation of the heart occurs early in the disease, objective measurement hereof with 123I-metaiodobenzylguanidine (MIBG) myocardial scintigraphy is considered a supportive criterion in the diagnosis of Parkinson’s disease [12]. MIBG scintigraphy allows the discrimination between PD and multiple system atrophy, since the peripheral autonomic nervous system is preserved in the latter [13, 14].

There is an increasing clinical and research interest in measuring, monitoring, and unravelling cardiovascular dysfunctions in PD patients. Subjective measures of cardiovascular symptoms (Fig. 1B) encompass patient-reported clinical scales such as the Non-Motor Symptoms Questionnaire (NMSQ) [15] and the Scales for Outcomes in Parkinson’s Disease-autonomic (SCOPA-aut) [16], which have sub-scores for cardiovascular symptoms. There are also clinician-rated assessment scales such as the Non-Motor Symptoms Scale (NMSS) [17], which includes a cardiovascular domain. The main objective tools (Fig. 1C) employed currently for measuring cardiovascular function include ambulatory or at-home monitoring of blood pressure and measures of heart rate and its variability (HRV). Orthostatic hypotension (OH) is classically defined as a drop in systolic blood pressure of at least 20 mm Hg or diastolic blood pressure of at least 10 mm Hg within three minutes of standing or head-up tilt [18]. Notably, varying cut-offs are used in clinical and investigational practice—including the diagnostic criteria for PD [12, 19]. In keeping with the neurogenic nature (i.e. failure of the baroreceptor reflex) of OH in PD, heart rate generally does not increase significantly during an orthostatic challenge [20]. This is different from non-neurogenic causes (e.g. dehydration, deconditioning, or heart failure) of OH, which are typically accompanied by a heart rate increase—effectively demonstrating an intact baroreceptor reflex [21]. A recent consensus panel of experts has proposed an increase in HR of < 15 beats per minute to support the diagnosis of neurogenic OH [22]. Moreover, recently an index has been validated which defines neurogenic OH as a heart rate to systolic blood pressure (HR/SBP) ratio of < 0.492 during the active standing test [23].

Heart rate variability (HRV) can be measured, to varying degrees of precision, with a standard electrocardiogram and more recently using wearables that have photoplethysmography sensors for detecting the interbeat interval. HRV measures the variation of time between successive heartbeats; it can be assessed with various analytical approaches, and the most commonly used are frequency domain (e.g. low frequency (LF; 0.04–0.15 Hz) and high frequency (HF; 0.15–0.4 Hz)) and time domain analysis [24] such as the Root Mean Square of Successive Differences (RMSSD) and the Standard Deviation of the N–N intervals (SDNN). Studies indicate that HF and RMSSD exhibit a degree of specificity for parasympathetic activity [25]. Conversely, the interpretation of LF is less straightforward; while initially viewed as a marker of sympathetic nervous system activity, recent research connects it to baroreflex function. The LF/HF power ratio serves as an indicator of sympatho-vagal balance. Finally, SDNN is defined as a measure of general cardiovascular health and when assessed over a 24 h period, SDNN is the clinical "gold standard" assessment for cardiovascular functions [25]. Heart rate variability (HRV) has been consistently used as a measure of autonomic functions in PD [26]. There is meta-analytic evidence that HRV is lower in patients with PD as compared to healthy controls even at an early stage of the disease [26, 27].

Deep brain stimulation (DBS) is a well-established treatment for motor symptoms and motor fluctuations in advanced PD [28]. The most commonly used targets of DBS for PD are the subthalamic nucleus (STN) and the globus pallidus pars interna (GPi). The ventral intermediate nucleus of the thalamus and the posterior subthalamic area have significant long-term benefit for tremor control but insufficient for other motor features of PD [29]. Finally, the pedunculopontine nucleus (PPN) is another target for DBS, but remains an investigational target. More recently, several studies have suggested a beneficial effect of DBS on non-motor PD symptoms overall and in some symptoms specifically (e.g. sleep and pain) [30]. The effect of DBS on cardiovascular PD symptoms, however, is unclear, with studies drawing conflicting conclusions [31, 32] especially with regard to the effect of DBS on HRV.

In this article, we aimed to systematically review the literature regarding the effect of DBS on cardiovascular indices in PD. We assessed (1) the effect of DBS on cardiovascular symptoms as subjectively reported by patients (i.e. via self-report questionnaires and clinician administered scales), and (2) the effect of DBS on cardiovascular functions evaluated with objective measures (i.e. measurements of blood pressure and heart rate variability).

## Methods

### Literature search

The present systematic review adhered to the recommendations of the Preferred Reporting Items for Systematic Reviews and Meta-Analyses (PRISMA) guidelines [33]. Each stage of this review was conducted according to the guidelines of the Cochrane Handbook of Systematic Reviews and Meta-Analyses.

The following inclusion criteria were used: studies involving PD patients treated with DBS (without prerequisites regarding DBS target); studies in which the effect of DBS on cardiovascular symptoms and/or on cardiovascular functions was reported (e.g. using clinical scales, measuring blood pressure (BP), heart rate or HRV; observational studies, randomised and non-randomised controlled trials and interventions; studies reported in English. Two investigators (FC, SH) assessed the eligibility of all full-text articles. When the 2 raters had disagreement in the inclusion of a study, a third rater (BS) was used to resolve the conflict.

A comprehensive search was performed on June 1st 2023, across two databases—PubMed (http://www.ncbi.nlm.nih.gov/pubmed/) and Medline (http://www.ovidsp.dc1.ovid.com)—to identify all articles published from inception until May 31th 2023. The following Medical Subject Headings (MESH) terms were used: “((Deep brain stimulation OR DBS) AND (cardiovascular symptoms OR nonmotor symptoms) AND (Parkinson disease OR PD) AND ((Non-Motor Symptoms Scale OR NMSS) OR (Non-Motor Symptoms Questionnaire OR NMSQ) OR (Scales for Outcomes in Parkinson’s Disease—Autonomic Dysfunction OR SCOPA-Aut))) OR ((Deep brain stimulation OR DBS) AND (hypotension OR hypertension OR blood pressure) AND (Parkinson disease OR PD)) OR ((Heart Rate Variability OR HRV) AND (deep brain stimulation OR DBS) AND (Parkinson disease OR PD))”. After performing the systematic search, we also manually checked the reference list of relevant articles and previous reviews to increase the sensitivity of our search strategy.

### Data extraction and analysis

The following data were extracted: study design, sample size, demographics (i.e. age, gender, disease duration, time since DBS surgery), DBS target (e.g., STN, GPi, PPN), method(s) used for clinical assessment of cardiovascular symptoms (if applicable), and method(s) and setting (i.e. in-hospital versus ambulatory, off-stimulation versus on-stimulation, off-medication versus on-medication) used for objective evaluation of cardiovascular functions (if applicable). If HRV measures were reported, HRV parameters both in the frequency and time domains were extracted. In case of incomplete data, additional information was requested from the corresponding authors.

To compare the effect of DBS on cardiovascular symptoms and BP/HRV measures across studies accounting for the varying methods of assessment (Fig. 1B and C), for each study, Cohen’s d effect sizes (postoperative versus preoperative, or on-stimulation versus off-stimulation) were calculated if mean and standard deviation were reported. Cohen’s d effect sizes were interpreted in the following ranges: unclear effect < 0.2, small effect 0.2–0.5, medium effect 0.5–0.8, and large effect > 0.8 [34]. For various settings, weighted Cohen’s d effect sizes were calculated factoring in the number of participants per study. To assess the relation between time since DBS surgery and pre to postoperative change in cardiovascular symptoms, linear regression was used with p-level defined at 0.05. Regarding the effect of DBS on blood pressure during orthostatic challenge, per setting, the weighted change in orthostatic blood pressure decrease was calculated. Analyses were performed with customised scripts using functions implemented in Matlab R2023a (Mathworks, Natick, MA).

## Results

**Fig. 2:**
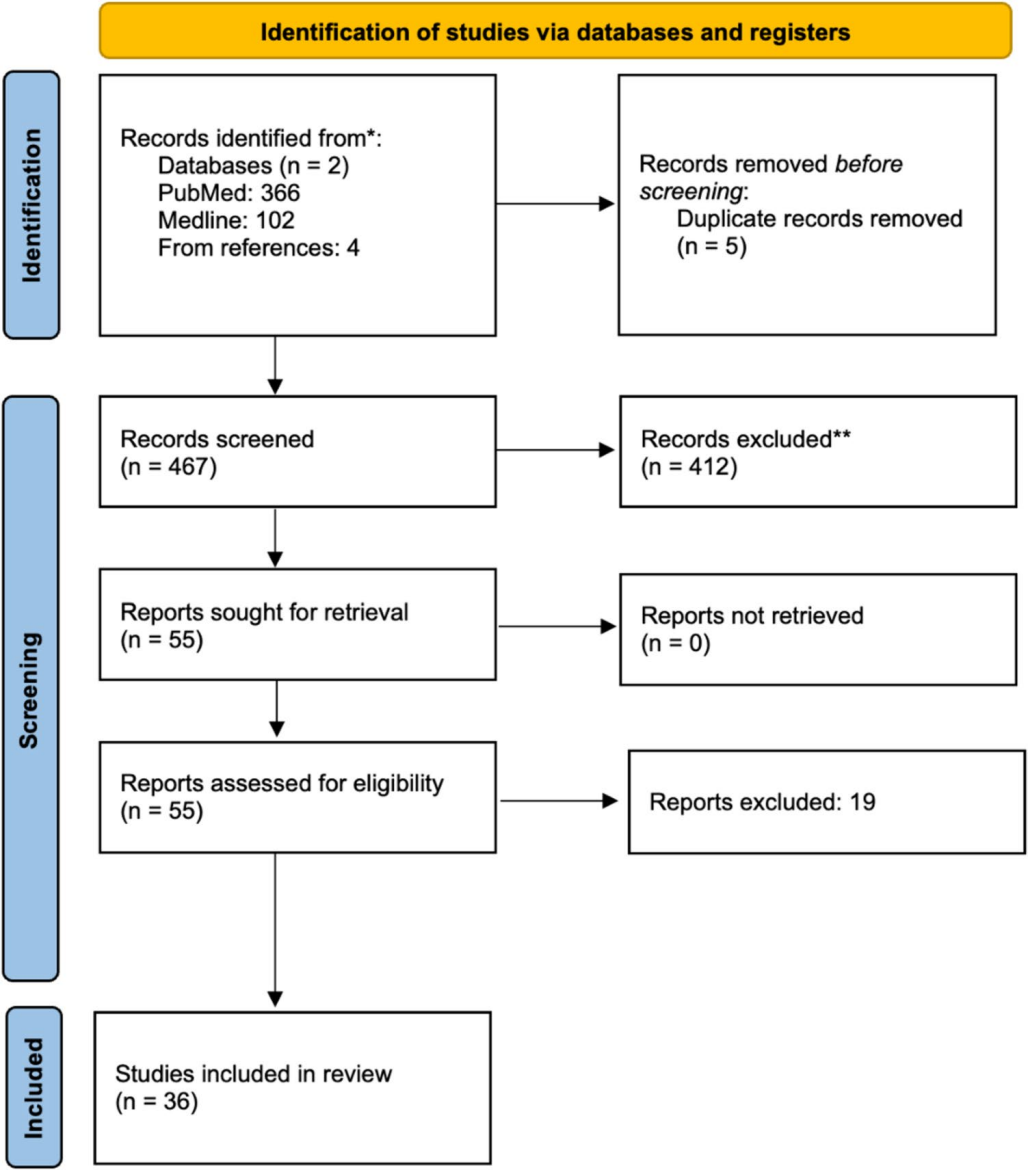
PRISMA flow diagram of the literature search

Our search strategy identified 472 articles (Fig. 2). After removing duplicates, the abstracts of 467 articles were screened and 55 of those were selected for full-text assessment. No additional studies were identified by reviewing the reference lists of these 55 studies. After full-text screening, 36 studies were included in the review. Preoperative and postoperative levodopa-equivalent daily dose (LEDD) was reported in 16 out of 36 studies. On average, after surgery, LEDD was reduced by 518 mg or 46.9%. Overall, studies comparing preoperative and postoperative evaluations mostly concerned prospective cohort studies, and studies comparing stimulation OFF with stimulation ON concerned within-subject studies—mostly without blinding.

### Effect of DBS on cardiovascular symptoms assessed by questionnaires/clinical scales

Seventeen studies evaluated the effect of DBS on cardiovascular symptoms, as assessed pre and postoperatively by self-reported or clinician-rated scales. The STN was the main target in all studies, except for one study on GPi. The total sample of these 17 studies was 885 PD patients with a mean age of 60.5 (SD 3.8) years and a mean disease duration of 10.8 (SD 1.4) years. The time between DBS surgery and postoperative assessment ranged from 1 to 36 months.

Estimation of Cohen’s d effect size was possible in 14 out of 17 studies (Table 1). The NMSS scale was the most frequent methodology, as it was employed in 12 out of 14 studies. In eleven of these 12 studies, NMSS was administered preoperatively and no later than one year after surgery. While all but one study reported a numeric decrease (i.e. improvement) in NMSS postoperatively, the improvement was reported as statistically significant in only five studies. The weighted Cohen’s d effect size across these 12 studies indicated a small improvement (Cohen’s d = − 0.26) of cardiovascular symptoms in the first year after surgery. Of note, the one study assessing both GPi-DBS and STN-DBS reported a moderate improvement (Cohen’s d = − 0.70) with GPi-DBS, which was larger than for STN-DBS [35]. Postoperative follow-up duration was longer than one year in three studies using NMSS. Two of these three studies reported a statistically significant worsening of cardiovascular symptoms compared to preoperatively, with weighted Cohen’s d across the three studies indicating a small worsening (Cohen’s d = 0.28). NMSQ was used in two out of 14 studies, and Scopa-Aut in one out of 14. These studies reported a numeric but not statistically significant improvement of cardiovascular symptoms, with a weighted Cohen’s d effect size suggesting a small improvement (Cohen’s d = − 0.20 for Scopa-Aut).

**Table 1:** Effect of DBS on cardiovascular symptoms

Study	*N*	Design	Age (years)—mean (SD)	Disease duration (years)—mean (SD)	Time since DBS surgery (m)	DBS target	Preoperative score—mean (SD)	Change compared to baseline		*p* value
NMSS cardiovascular subdomain (two severity-frequency questions; 0–24)								Absolute change	Cohen’s d	
Kurcova et al. 2018	24	PCS	62.3 (6.0)	8.0 (3.9)	1	STN	1.7 (3.2)	− 1.50	− 0.65	0.021*
Kurcova et al. 2018	24	PCS	62.3 (6.0)	8.0 (3.9)	4	STN	1.7 (3.2)	− 1.30	− 0.54	0.063
Dafsari et al. 2018	67	PCS	62.3 (7.8)	10.9 (4.8)	5	STN	1.9 (3.4)	− 0.60	− 0.21	0.018*
Dafsari et al. 2015	60	PCS	61.6 (7.8)	10.4 (4.2)	6	STN	1.6 (2.3)	− 0.58	− 0.29	0.096
Ricciardi et al. 2018	18	PCS	49.6 (7.6)	11.4 (3.6)	6	STN	1.4 (2.8)	− 0.94	− 0.46	0.100
Dafsari et al. 2019	98	PCS	61.5 (9.5)	10.7 (4.8)	6	STN	1.1 (2.0)	− 0.20	− 0.11	0.466
Petry-Schmelzer et al. 2019	91	PCS	62.7 (7.9)	9.9 (4.6)	6	STN	2.2 (3.4)	− 0.89	− 0.30	0.075
Dafsari et al. 2020	30	PCS	58.5 (12.4)	10.4 (5.6)	6	STN	1.0 (2.0)	0.00	0.00	0.749
Dafsari et al. 2020	18	PCS	58.1 (9.1)	11.0 (4.0)	6	GPi	2.1 (3.0)	− 2.10	− 0.70	0.020*
Hwynn et al. 2011	10	PCS	66.1 (7.8)	9.9 (3.0)	12	STN (9), GPi (1)	N/A	− 1.90	N/A	0.020*
Cury et al. 2014	41	PCS	60.0 (10.4)	15.0 (7.6)	12	STN	6.2 (6.3)	− 0.82	− 0.13	0.322
Deli et al. 2015	25	PCS	55.9 (8.7)	11.0 (4.8)	12	STN	4.3 (4.1)	− 1.80	− 0.50	0.049*
Ricciardi et al. 2018	18	PCS	49.6 (7.6)	11.4 (3.6)	12	STN	1.4 (2.8)	− 0.78	− 0.33	0.100
Total ≤ 12 m	500							−0.72	− 0.26	
Ricciardi et al. 2018	18	PCS	49.6 (7.6)	11.4 (3.6)	24	STN	1.4 (2.8)	− 0.20	− 0.08	0.100
Dafsari et al. 2018	67	PCS	62.3 (7.8)	10.9 (4.8)	24	STN	1.9 (3.4)	0.50	0.15	0.018*
Jost et al. 2020	38	PCS	61.3 (8.5)	10.8 (4.9)	36	STN	0.8 (1.3)	1.60	0.69	0.012*
Total > 12 m	123							0.74	0.28	
NMSQ cardiovascular subdomain (two yes/no questions; 0–2)								Absolute change	Cohen’s d	
Hwynn et al. 2011	10	PCS	66.1 (7.8)	9.9 (3.0)	12	STN (9), GPi (1)	N/A	− 0.50	N/A	0.230
Nazzaro et al. 2011	24	RCS	64.2 (6.5)	10.6 (3.5)	12	STN	0.9 (N/A)	− 0.25	N/A	N/A
Total ≤ 12 m	34							-0.32	N/A	
SCOPA-Aut cardiovascular subdomain (three severity-frequency questions; 0–9)								Absolute change	Cohen’s d	
Bjerknes et al. 2020	58	PCS	62 (N/A)	11 (N/A)	3	STN	0.8 (1.1)	− 0.20	− 0.20	0.172
Bjerknes et al. 2020	58	PCS	62 (N/A)	11 (N/A)	12	STN	0.8 (1.1)	− 0.20	− 0.19	0.172
Total ≤ 12 m	58							− 0.20	− 0.20	

This table summarizes studies (*n* = 14) identified in the systematic literature review that measured cardiovascular symptoms preoperatively and postoperatively by means of clinical scales. Three studies were not included in this table since data required for Cohen’s d estimation were not available—these studies are described in the main text. The table is subdivided in studies using the Non-Motor Symptoms Scale (NMSS), Non-Motor Symptoms Questionnaire (NMSQ) and Scales for Outcomes in Parkinson’s Disease—Autonomic (SCOPA-Aut). Division is also made between studies with the postoperative assessment occurring within 12 months after surgery, and those later than 12 months. Absolute changes and Cohen’s d effect sizes were calculated based on the preoperative (displayed in table) and postoperative (not displayed in table) score on the respective scale. Negative change scores or negative Cohen’s d values indicate an improvement at the postoperative evaluation compared to preoperatively. Below each subdivision of studies, weighted (i.e. average but weighted according to amount of participants per study) absolute change scores and Cohen’s values are reported. P-values concern those reported in the retrospective study, with reported statistical significance indicated with an asterisk

*DBS* deep brain stimulation, *GPi* globus pallidus pars interna, *PCS* prospective cohort study, *RCS* retrospective cohort study, *SD* standard deviation, *STN* subthalamic nucleus

**Fig. 3:**
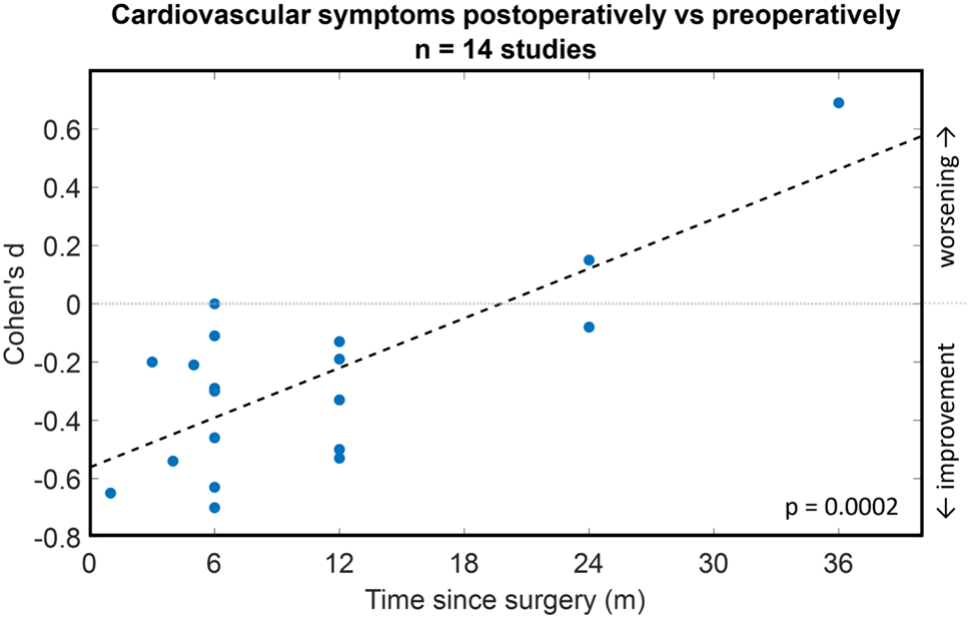
Effect of DBS on cardiovascular aspects of Parkinson’s disease over time. 17 studies assessed the effect of DBS on cardiovascular symptoms by comparing preoperative and postoperative questionnaires or scales. Cohen’s d effect sizes could be calculated for 14 out of 17 studies, with negative Cohen’s d values indicating an improvement compared to preoperatively, and positive Cohen’s d values indicating a worsening. In this graph, for each of these 14 studies, the respective Cohen’s d value is plotted against the time between surgery and postoperative assessment. Note that some studies included multiple assessments over time. Relation between Cohen’s d effect size and time since surgery was assessed by linear regression (dashed line), demonstrating a significant (*p* = 0.0002) correlation

Considering all 14 studies employing either NMSS, NMSQ, or Scopa-Aut, there was a significant (p = 0.0002) relation between follow-up duration (i.e. time since DBS surgery) and Cohen’s d effect size of the pre to postoperative change in cardiovascular symptoms (Fig. 3): the longer the follow-up duration, the smaller the postoperative improvement in cardiovascular symptoms.

Three studies were not included in Table 1 since data required for Cohen’s d estimation were not available. Although one study (n = 36) reported an improvement in cardiovascular symptoms measured via NMSS in the first year after surgery [36], no statistically significant change was observed in the other two studies with a sample of n = 190 (the largest sample size of all studies) [37] and n = 57 [32].

One study also included a non-operated control group (n = 38 in both the DBS and the control group), and found no between-group difference in the change from baseline to follow-up assessment at 3 years NMSS cardiovascular subdomain [38].

### Effect of DBS on blood pressure during orthostatic challenge

Ten studies evaluated the effect of DBS on BP during an orthostatic challenge (Table 2). STN-DBS was employed in nine, and PPN-DBS in two studies. The ten studies encompassed 149 PD patients with a mean age of 61.3 (SD 3.8) years and a mean disease duration of 14.9 (SD 2.7) years. The time between DBS surgery and postoperative assessment ranged from 1 to 70 months. Tilt Table Testing (or Head Up Test) was the most frequently employed method, and the majority of studies were conducted in the on-medication state.

**Table 2:** Effect of DBS on blood pressure during an orthostatic challenge

Study	*N*	Design	Age (year)—mean (SD)	Disease duration (years)—mean (SD)	Time since DBS surgery (m)	DBS target	Blood pressure method	Medication state	Orthostatic SBP dip (mmHg)—mean (SD)		
Postoperative vs preoperative									Preop	Postop	Change
Holmberg et al. 2005	11	PCS	57.9 (5.1)	17.2 (4.8)	12	STN	HUT	On	N/A	N/A	No difference
Trachani et al. 2012	24	PCS	62.1 (9.4)	12.8 (6.5)	6	STN	Sit and stand	On	N/A	N/A	Resolution of OH in 8/11
Tateno et al. 2015	8	PCS	65.3 (5.5)	12.9 (2.8)	1–24	STN (7), GPi (1)	HUT	N/A	51.6 (10.8)	17.3 (11.6)	− 34.4 (8.7)
Total STN/GPi	8										− 34.4
Postoperative: ON-stim vs OFF-stim									OFF-stim	ON-stim	Change
Priori et al. 2001	9	WSS, B-	59.0 (8.5)	N/A	3–24	STN	HUT	On	N/A	N/A	No difference
Stemper et al. 2006	14	WSS, B-	58.1 (5.8)	N/A	4–10	STN	HUT	On	11.0 (N/A)	17.7 (N/A)	6.7 (N/A)
Ludwig et al. 2007	14	WSS, B-	57.7 (2.8)	N/A	N/A	STN	HUT	On	N/A	N/A	No difference
Sverrisdottir et al. 2014	5	WSS, B-	N/A	11.1 (6.2)	N/A	STN	HUT	On	N/A	N/A	Variable response
Li et al. 2017	26	WSS, B-	68.7 (5.4)	15.7 (4.0)	7–18	STN	HUT	Off	11.2 (27.5)	12.5 (20.3)	1.3 (N/A)
Fabbri et al. 2017	32	WSS, B-	62.5 (13.3)	18.7 (5.1)	40–70	STN	Supine and stand	Off	6.0 (N/A)	14.0 (N/A)	8.0 (N/A)
Total STN/GPi	72										5.3
Hyam et al. 2019	6	WSS, B +	60.3 (N/A)	15.7 (N/A)	12–34	PPN	HUT	On	17.2 (4.6)	8.3 (3.4)	− 8.9 (N/A)
Sverrisdottir et al. 2014	1	WSS, B-	N/A	25 (N/A)	N/A	PPN	HUT	On	N/A	N/A	No difference
Total PPN	6										− 8.9

This table summarizes studies (*n* = 10) identified in the systematic literature review that assessed the effect of DBS on objectively measured blood pressure during an orthostatic challenge. The table is subdivided in studies that compared a preoperative with postoperative assessment, and those that performed only postoperative assessments but compared the off-stimulation with on-stimulation measurement. Division is also made between studies with conventional DBS targets (i.e. STN or GPi) and studies with other targets (e.g. PPN). The last three columns relate to ‘orthostatic systolic blood pressure (SBP) dip’, that is the absolute difference of the SBP in standing compared to lying/sitting position. If applicable data were available in the study, change scores were reported in the last column—with negative values indicating an improvement (i.e. less orthostatic SBP dip postoperatively or with stimulation on). Per subdivision, weighted (i.e. accounting for number of participants per study) averages were calculated

*B* blinding (−/ + absent/present), *DBS* deep brain stimulation, *GPi* globus pallidus pars interna, *HUT* head-up test (i.e. tilt table testing), *PCS* prospective cohort study, *PPN* pedunculopontine nucleus, *SBP* systolic blood pressure, *SD* standard deviation, *STN* subthalamic nucleus, *WSS* within-subjects study

Three studies evaluated changes in BP during an orthostatic challenge before and after DBS (Table 2). One study, mostly including patients without orthostatic hypotension, reported no change [39]. Two studies included patients with OH and showed 62% [40] and 72% [41] improvement in the number of people reporting OH after DBS as compared to preoperatively.

Six studies evaluated the acute effect of STN-DBS on blood pressure during an orthostatic challenge (Table 2). Despite heterogeneity in the time since surgery (between 3 and 70 months) and the dopaminergic medication state, no obvious differences in orthostatic systolic blood pressure dip were noted with stimulation ON compared to stimulation OFF. Across these studies, the weighted change in orthostatic systolic blood pressure dip was 5.3 mmHg (i.e. worse). In one study, however, systolic blood pressure dip upon standing was smaller (i.e. improved) when stimulation was delivered in the dorsal STN selectively [42].

The acute effect of PPN-DBS on orthostatic systolic blood pressure dip was assessed in two studies. Whereas no difference was observed in one patient [42], a case series of 6 patients reported a significant improvement of 8.9 mmHg [43].

Two studies included a non-operated control group [39, 44]. Despite limited data, orthostatic systolic blood pressure dips were more pronounced in patients on optimal pharmacotherapy than in those who underwent STN-DBS.

Two studies performing supine blood pressure measurements [45] and during a cold pressor test [46] were not included in Table 2 because no orthostatic challenge has been performed. These studies reported no difference between on-stimulation and off-stimulation assessments.

### Effect of DBS on HRV

Twelve studies assessed the effect of DBS on HRV in PD patients (Table 3). The STN was the DBS target in 11 studies, and the PPN in one study [43]. Altogether, these 12 studies encompassed 196 PD patients with a mean age of 61.3 (SD 3.6) years and a mean disease duration of 14.8 (SD 3.9) years. Measurements were mostly performed while the patients were resting, and had in general a short (approximately 5 min) duration—only one study had a 24-h duration [47]. Around half of the studies were performed in the off-medication state, and half in the on-medication state. Frequency domain HRV measures (mostly LF and HF) were most frequently employed. Incomplete reporting precluded Cohen’s d effect size calculation in many studies, hence study interpretation in this section is largely qualitative.

**Table 3:** Effect of DBS on heart rate variability

Study	*N*	Design	Age (years)—mean (SD)	Disease duration (years)—mean (SD)	Time since DBS surgery (m)	DBS target	Con-dition	Medica- tion state	Recor-ding duration	Change—Cohen’s d					
										LF	HF	LF/HF	TP	SDNN	RMSSD
Postoperative vs preoperative															
Azevedo et al. 2010	16	PCS	57.0 (9.1)	N/A	2–13	STN	Rest	Off	N/A	N/A	− 0.41	N/A	N/A	N/A	N/A
Sumi et al. 2012	28	PCS	62.4 (7.4)	22 (N/A)	0.5	STN	Rest	Off	5 min	N/A	− 0.01	N/A	N/A	N/A	N/A
Chen et al. 2011	16	PCS	63.0 (N/A)	8.5 (2.6)	9–32	STN	Rest	On	5 min	2.90*	=	N/A	N/A	N/A	N/A
Trachani et al. 2012	24	PCS	62.1 (9.4)	12.8 (6.5)	6	STN	Rest	On	5 min	− 0.55	0.08	↑	− 0.47	N/A	N/A
Sumi et al. 2012	28	PCS	62.4 (7.4)	22 (N/A)	0.5	STN	Rest	On	5 min	N/A	1.94	N/A	N/A	N/A	N/A
Erola et al. 2006	14	PCS	57.0 (9.0)	12.0 (7.0)	12	STN	24 h routine	On	24 h	− 0.23	− 0.02	N/A	N/A	− 0.06	N/A
Postoperative: ON-stim vs OFF-stim															
Benedetti et al. 2004	10	WSS, B-	61.6 (8.0)	16.1 (4.4)	0	STN (dorsal)	Rest†	Off	3 min	↑	↑	N/A	N/A	N/A	N/A
Benedetti et al. 2004	10	WSS, B-	61.6 (8.0)	16.1 (4.4)	0	STN (ventral)	Rest†	Off	3 min	↑	=	N/A	N/A	N/A	N/A
Lannotte et al. 2005	20	WSS, B +	61.8 (N/A)	15.7 (N/A)	0	STN (dorsal)	Rest†	Off	3 min	↑	N/A	N/A	N/A	N/A	N/A
Lannotte et al. 2005	20	WSS, B +	61.8 (N/A)	15.7 (N/A)	0	STN (ventral)	Rest†	Off	3 min	↑‡	N/A	N/A	N/A	N/A	N/A
Sumi et al. 2012	28	WSS, B-	62.4 (7.4)	22 (N/A)	0.5	STN	Rest	Off	5 min	N/A	− 0.56	N/A	N/A	N/A	N/A
Li et al. 2017	26	WSS, B-	68.7 (5.4)	15.7 (4.0)	12	STN	Rest	Off	5 min	=	=	− 0.31	N/A	N/A	N/A
Liu et al. 2013	8	WSS, B-	66.1 (7.4)	N/A	20–32	STN	Rest (sleep)	Off	1 h	↓	↓	↑	↓	N/A	N/A
Ludwig et al. 2007	14	WSS, B-	57.7 (2.8)	N/A	N/A	STN	Rest	On	N/A	N/A	N/A	N/A	N/A	N/A	=
Sumi et al. 2012	28	WSS, B-	62.4 (7.4)	22 (N/A)	0.5	STN	Rest	On	5 min	N/A	− 0.23	N/A	N/A	N/A	N/A
Li et al. 2017	26	WSS, B-	68.7 (5.4)	15.7 (4.0)	12	STN	HUT	Off	5 min	=	=	=	N/A	N/A	N/A
Stemper et al. 2006	14	WSS, B-	58.1 (5.8)	N/A	4–10	STN	HUT	On	5 min	=	=	=	N/A	N/A	N/A
Li et al. 2017	26	WSS, B-	68.7 (5.4)	15.7 (4.0)	12	STN	DB	Off	5 min	=	=	=	N/A	N/A	N/A
Ludwig et al. 2007	14	WSS, B-	57.7 (2.8)	N/A	N/A	STN	DB	On	N/A	N/A	N/A	N/A	N/A	N/A	=
Hyam et al. 2019	6	WSS, B +	60.3 (4.1)	15.6 (4.2)	18–30	PPN	Valsalva	On	3 min	↑	↓	↑	N/A	N/A	N/A

This table summarizes studies (*n* = 12) identified in the systematic literature review that assessed the effect of DBS on measures of heart rate variability (HRV). The table is subdivided in studies that compared a preoperative with postoperative assessment, and those that performed only postoperative assessments but compared the off-stimulation with on-stimulation measurement. The last six columns relate to the change in HRV (as assessed by one or more out of six HRV measures) on-stimulation/postoperative compared to off-stimulation/preoperative. Change scores constitute Cohen’s d effect sizes, calculated based on reported values. Negative change scores indicate a decrease in the HRV measure with stimulation compared to without stimulation. If exact values were not reported, directionality of changes is reported which is based on qualitative interpretation of reported data/graphs

*B*, blinding (−/ + absent/present), *DB* deep breathing, *DBS* deep brain stimulation, *LF* low frequency, *HF* high frequency, *HUT* head-up test, *PCS* prospective cohort study, *PPN* pedunculopontine nucleus, *RMSSD* root mean square of successive differences, *SDNN* standard deviation of the N–N intervals, *STN* subthalamic nucleus, *TP* total power, *WSS* within subjects-study

*only in those with a ‘good response’, †intraoperatively, ‡ only when patients were unblinded to stimulation, = unchanged

Five out of 12 studies made a comparison between preoperative and postoperative HRV values, and reported varying outcomes. Two studies showed no significant change in HRV after STN-DBS [47, 48]. One study reported an increase in LF but not HF power, however only in patients who had a good motor outcome after surgery (i.e. decrease in the UPDRS total score > 50%) [49]. These data are, however, conflicted by one study reporting decreased LF [50], and another study reporting increased HF [41].

Eight studies evaluated the acute effect of DBS on HRV measures in PD patients. Whereas three studies showed no significant change [43, 44, 50, 51], differences between off-stimulation and on-stimulation were reported in the other studies. Although the directionality and magnitude of these differences varied considerably across studies, there are some commonalities. Two intraoperative studies assessed the contribution of stimulation voltage and stimulation location to the effect of DBS on HRV. One study observed an increase in LF with stimulation, but only upon higher stimulation voltages when stimulation was delivered in a blinded (‘unaware’) way in the most ventral contacts in the motor STN [52]. In another study from the same group, the increase of LF upon stimulation of the most ventral contact in the motor STN was influenced by the awareness of the patient to the stimulation—suggesting possible limbic influences when stimulating this portion of the STN possibly because of the spread of current to the medial STN [53]. This notion is further corroborated by another study—conducted during sleep—demonstrating a relation between the distance of clinically effective stimulation from the structures implicated in sympathetic regulation (like limbic pathways and zona incerta) and the increase of LF/HF ratio [54]. Opposed to this DBS-induced LF/HF ratio increase, another study reported LF/HF ratio to decrease with stimulation [55].

Four studies evaluated the acute effect of DBS on HRV during active conditions. Tilt Table Testing was employed in two studies, both of which demonstrated no effect of DBS on HRV parameters [51, 55]. In line herewith, DBS was reported not to affect HRV when assessed during a Valsava manoeuvre [43] or controlled deep breathing [44, 55].

## Discussion

The role of DBS on motor symptoms in people with PD is well defined and its effect on non-motor symptoms has recently gained interest [28, 56]. In the present study we systematically reviewed the evidence regarding the effect of DBS on cardiovascular symptoms and objective measures of cardiovascular functioning in PD patients.

Most of the studies included patients with STN-DBS, a small number of studies have also looked at the effect of GPi-DBS or PPN-DBS on cardiovascular measures in PD.

Overall, these studies suggest that, when exploring the effect of DBS on cardiovascular symptoms using questionnaires or clinical scales, there is a short-term improvement in symptom severity in the first year after DBS. The magnitude of the improvement is, however, small, so clinical relevance remains unclear. Interestingly, the postoperative improvement in cardiovascular symptoms seems to disappear with longer DBS duration, with even worsening of symptoms compared to preoperatively.

This temporal response of symptoms to DBS can be related to a number of factors including the change in medications after surgery and the underlying disease progression. Indeed, levodopa and dopaminergic medications in general are known to have an effect on cardiovascular functions in PD [57], with higher doses having a more detrimental effect than lower doses [58]. The reported short-term improvement in cardiovascular symptoms after DBS may reflect the decrease in total levodopa equivalent daily dose (46.9% reduction in the included studies), however only scanty data are available and there is no control for this variable in the vast majority of the studies.

Moreover, the loss of benefit and even the worsening of cardiovascular symptoms in the studies with longer follow-up is likely a reflection of disease progression. Indeed, although autonomic dysfunction can occur at any stage of the disease, a correlation between disease duration and worsening of autonomic functions has been reported in PD [59].

One study suggested a significant improvement in cardiovascular symptoms, using a self-report questionnaire, in PD patients after 6 months of GPi-DBS and not STN-DBS [35]. Of note, GPi-DBS patients had more severe baseline cardiovascular symptoms, in line with real-world practice, where GPi-DBS is proposed for frailer elderly patients, with poorer cognition and more comorbid conditions, including orthostatic hypertension [60]. Although a moderate effect size is reported by the authors, validation of this single report is needed. No study has evaluated this effect of GPi using objective measures of blood pressure or HRV.

The effect of DBS on blood pressure and HRV is more complex. We evaluated both longitudinal studies comparing preoperative with postoperative assessment, and cross-sectional studies performing postoperative assessments comparing off-stimulation versus on-stimulation measurement.

The longitudinal studies assessing PD patients before and after DBS suggest that STN-DBS can reduce objectively measured orthostatic hypotension. This again might be in relation to the reduction of dopaminergic medications that is usually achieved with STN-DBS, as a detrimental effect of levodopa and other dopaminergic medication on blood pressure regulation has been demonstrated [61, 62].

Another more speculative explanation could be that the reduction in orthostatic hypotension might result from the spread of the electrical stimulation to neighbouring structures of the STN that are implicated in the regulation of blood pressure, such as, for example the posterior subthalamic area and fibres involved in the sympathetic control. Whereas stimulation of the STN itself does not seem to reduce orthostatic hypotension, the role of PPN in regulating cardiovascular functions has been suggested in one of the studies included in this review. This small study suggested a significant improvement in orthostatic hypotension and in the cardiovascular response during the Valsalva manoeuvre compared to without PPN stimulation [43]. The mechanisms through which the PPN regulates cardiovascular functions remain elusive. A direct effect on the peripheral vascular tone and on myocardial contractility as well as a role in the neural control of the baroreflex activation have been suggested [43].

Cross-sectional studies evaluating the effect of STN-DBS on blood pressure and HRV, suggest an effect of the intensity of the electrical stimulation (amplitude) and the exact location of the stimulation within the STN. Specifically, high amplitude and stimulation in the inferomedial part of the STN were associated with a stronger effect on blood pressure and HRV. This could be mediated by the spread of current to areas involved in the central regulation of the autonomic nervous system, and supports a role of the medial STN in sympathetic control, through its limbic connections [63]. An important limitation of the studies included in the present review, however, is a lack of information on the precise location of the DBS electrodes and the volume of tissue activated.

In summary, the available longitudinal studies show an overall improvement of cardiovascular symptoms, mainly orthostatic hypotension, in the short-term after STN-DBS. This effect dissipates over time, possibly reflecting a beneficial effect of dopaminergic medication reduction after surgery and a detrimental effect of ongoing disease progression on autonomic functions. This is also supported by the results of cross-sectional studies showing little or no effect of STN-DBS (stimulation ON versus stimulation OFF) on more objective measures such as blood pressure and HRV during a challenge. Interestingly, some preliminary reports suggest that when stimulation is targeted towards or spreads to structures in the vicinity of the STN (e.g. hypothalamus, zona incerta), a direct beneficial modulation of cardiovascular functions could be achieved.

To better understand and optimize the effect of DBS on cardiovascular functions in PD, future studies will need to evaluate stimulation at conventional targets (i.e. STN and GPi—the latter currently underrepresented and showing promise) and more investigational ones (e.g. PPN). Since the questionnaires used in the available studies evaluate almost exclusively orthostatic hypotension, effort needs to be made to include other cardiovascular symptoms (e.g. postprandial hypotension and supine hypertension) in the assessments. Objective measures, incorporated in a well-designed protocol possibly including stressors (e.g. orthostatic challenge, Valsalva etc.), should supplement subjective assessments. A number of confounding factors should be taken into account in the future, including the role of medications (both those for PD and those prescribed for other conditions), the effect of motor and non-motor symptoms, and the effect of disease duration. Also, more objective, experimental ON/OFF stimulation studies are needed, with rigorous methods including blinded design, testing multiple stimulation amplitudes and looking at the exact spatial spread of direct neural activation in response to electrical stimulation [64].

A better understanding of the anatomical and functional interplay between DBS and cardiovascular systems will be required for the development of targeted modulation of cardiovascular functions in PD via DBS.

## Conclusions

In conclusion, although the effect of DBS on motor symptoms is well-established, its effect on autonomic nervous system and specifically on cardiovascular functions remains unclear. Here, we find evidence supportive of an acute benefit on cardiovascular symptoms in the first year post-operatively, but these benefits do not appear sustained as disease progresses. Future studies are encouraged to investigate this relevant topic that can have a strong clinical impact.
